# Specific inhibition of p110α subunit of PI3K: putative therapeutic strategy for *KRAS* mutant colorectal cancers

**DOI:** 10.18632/oncotarget.11843

**Published:** 2016-09-02

**Authors:** Maria Sofia Fernandes, Soraia Melo, Sérgia Velho, Patrícia Carneiro, Fátima Carneiro, Raquel Seruca

**Affiliations:** ^1^ Instituto de Investigação e Inovação em Saúde/Institute for Research and Innovation in Health (i3S), University of Porto, Porto, Portugal; ^2^ Institute of Molecular Pathology and Immunology of the University of Porto (IPATIMUP), Porto, Portugal; ^3^ Faculty of Medicine, University of Porto, Porto, Portugal; ^4^ Department of Pathology, Centro Hospitalar São João, Porto, Portugal

**Keywords:** colorectal cancer, targeted therapies, PI3K p110α, KRAS, BYL719

## Abstract

Colorectal cancer (CRC) is a leading cause of cancer mortality worldwide. It is often associated with activating mutations in *KRAS* leading to deregulation of major signaling pathways as the RAS-RAF-MAPK and PI3K-Akt. However, the therapeutic options for CRC patients harboring somatic *KRAS* mutations are still very limited. It is therefore urgent to unravel novel therapeutic approaches for those patients. In this study, we have awarded PI3K p110α a key role in CRC cells harboring *KRAS/PIK3CA* mutations or *KRAS* mutations alone. Specific silencing of PI3K p110α by small interfering RNA (siRNA) reduced viability and induced apoptosis or cell cycle arrest. In agreement with these cellular effects, PI3K p110α silencing led to alterations in the expression levels of proteins implicated in apoptosis and cell cycle, namely XIAP and pBad in *KRAS/PIK3CA* mutant cells and cyclin D1 in *KRAS* mutant cells. To further validate our data, a specific PI3K p110α inhibitor, BYL719, was evaluated. BYL719 mimicked the *in vitro* siRNA effects on cellular viability and on the alterations of apoptotic- and cell cycle-related proteins in CRC mutant cells. Overall, this study demonstrates that specific inhibition of PI3K p110α could provide an alternative therapeutic approach for CRC patients, particularly those harboring *KRAS* mutations.

## INTRODUCTION

Colorectal cancer (CRC) remains one of the most common malignancies and leading causes of cancer mortality worldwide [1]. Developing as a multistep process, CRC results from the accumulation of both genetic and epigenetic alterations in a number of oncogenes and tumor suppressor genes [2]. It is well established that deregulation of the EGFR signaling is frequent in CRC [3]. Indeed, our group and others have demonstrated that mutations in genes downstream of EGFR, namely in *KRAS*, *BRAF* and *PIK3CA* (the gene coding for p110α, the catalytic subunit of PI3K) are of particular relevance in microsatellite instable (MSI) and microsatellite stable (MSS) molecular subsets of CRC [4, 5]. Approximately 30%-40% of CRC patients do harbor a mutation in *KRAS*, while mutations in *BRAF* are generally found in about 15% of CRC patients [4–8]. Of significance is the fact that *KRAS* and *BRAF* mutations are preferentially found as alternative molecular alterations and thus are not frequently observed in the same tumor suggesting that KRAS and BRAF have distinct roles in the development and progression of CRC [5, 9]. In contrast, *PIK3CA* mutations, which are present in about 15% of CRC patients, occur concomitantly with *KRAS* or *BRAF* mutations [4, 8, 10]. Notably, mutations in these genes lead to constitutive activation of major signaling pathways downstream of EGFR with effects in the regulation of proliferation, growth, survival, invasion and cancer metabolism [3, 4].

To date, a number of targeted biological therapies have been developed to improve CRC patient survival [11]. Indeed, patients with metastatic CRC (mCRC) can be offered anti-VEGF therapy using bevacizumab, namely in combination with chemotherapy regimens, which has proven some clinical efficacy [12]. In particular, EGFR-targeted therapies have received much attention with cetuximab and panitumumab, two anti-EGFR antibodies, currently approved for the treatment of patients with mCRC [13]. Regrettably, CRC patients harboring somatic *KRAS* mutations are not eligible to such therapies, and indeed, *KRAS* mutations have been recognized as predictive markers of resistance to anti-EGFR therapies [13–15]. In addition, recent recommendations encourage CRC patients to be tested for *NRAS* mutations prior to EGFR targeted therapies, as these are now regarded as predictors of resistance, though *NRAS* mutations are only observed in about 2% of CRC patients [15, 16]. Therefore, this clinical limitation calls for the urgent need to further identify new therapeutic strategies for CRC patients, particularly those with KRAS activation by oncogenic mutations. KRAS is a member of the RAS superfamily of GTPases that is important in controlling diverse biological functions including cell proliferation, differentiation, survival and death [17]. The classical RAS signal transduction pathway involves sequential phosphorylations of the serine/threonine kinase RAF, MEK1/2 and ERK1/2, ultimately modulating other molecules and regulating the distinct biological functions [18]. In addition, RAS is also known to activate other signaling cascades namely the PI3K signaling pathway [19, 20]. In turn, PI3Ks are a rather ubiquitous family of lipid kinases able to phosphorylate the 3′-hydroxyl group of phosphatidylinositol and phosphoinositides, and these lipid products act as second messengers to trigger a multitude of signalling cascades with impact in key mechanisms as survival, differentiation and metabolism [20, 21].

The PI3Ks are grouped into three classes (I-III), with distinct structures and substrate specificities but class IA have received much attention as they have been implicated in many human cancers. Class IA PI3Ks, able to phosphorylate phosphatidylinositol (4,5)-biphosphate (PIP2), converting it to phosphatidylinositol (3,4,5)-triphosphate (PIP3), are composed of a heterodimer of a p85 regulatory subunit and a p110 catalytic subunit [20, 21]. Subsequent activation of the serine/threonine kinase Akt, the major PI3K target, leads to phosphorylation of additional downstream molecules that will ultimately modulate the many functions of the PI3K signaling cascade [22]. In CRC, the p110α subunit of PI3K has been the main focus of research as it is often mutated in these patients [4, 5].

Notably, pan-PI3K inhibitors, which inhibit all isoforms of class I PI3K, or dual PI3K/mTOR inhibitors have been evaluated in many studies. More specifically, early preclinical *in vitro* and *in vivo* data with the class I PI3K inhibitor LY294002 had shown anti-tumor activity in CRC cells [23]. Other drugs were subsequently developed with improved features for clinical evaluation including BKM120, XL-147, XL-765 and BEZ235 [24]. However, and despite ongoing clinical trials with some of these drugs, no substantial benefit has yet been observed and CRC patients are still awaiting successful alternative therapies to be approved.

Therefore, this study aimed at evaluating the cellular effects of PI3K p110α specific inhibition in human CRC cell lines with *KRAS* and *KRAS/PIK3CA* mutations. As a first approach, targeting of PI3K p110α was achieved by small interfering RNA (siRNA) and functional responses investigated. Interestingly, a recently developed PI3K p110α inhibitor, BYL719, is currently under clinical evaluation but little is known on the mechanisms of PI3K p110α inhibition [24–26]. Thus, investigation on the effects of BYL719 was also pursued in this study. Our data strongly indicates that CRC cells harboring alterations in KRAS and/or PI3K are sensitive to PI3K p110α inhibition providing an alternative therapeutic approach for *KRAS* mutant CRC patients who are currently excluded from EGFR-targeted therapies.

## RESULTS

The signaling pathways RAS-RAF-MAPK and PI3K-Akt are considered attractive target cascades for CRC therapy. Therefore, a panoply of inhibitors have been developed, namely for RAF, MEK1/2, PI3K, Akt, mTOR, while KRAS has been proved difficult to target pharmacologically. In this study, we aimed to elucidate the potential benefit of targeting the PI3K signaling pathway, more specifically the PI3K p110α subunit, in CRC cells harboring mutations in *KRAS* and/or *PIK3CA*. For this purpose, human CRC cell lines were selected to closely mimic the mutational pattern found in CRC patients regarding hotspot mutations in *KRAS* and *PIK3CA*: HCT116 (KRAS^G13D^, PIK3CA^H1047R^) and SW480 (KRAS^G12V^).

### Specific inhibition of PI3K p110α decreases viability in *KRAS* and *KRAS/PIK3CA* mutant CRC cells

**Figure 1:**
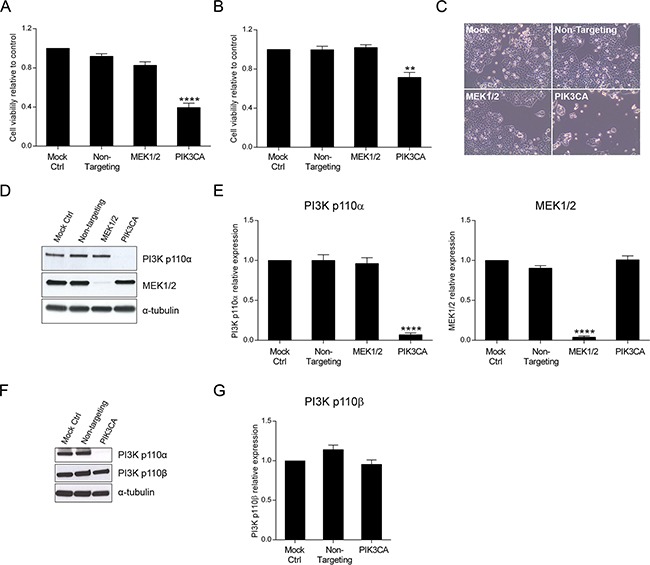
Inhibition of PI3K p110α decreases viability in HCT116 and SW480 CRC cells The effect of siRNA-mediated targeted depletion of *MEK1/2* and *PIK3CA* on cellular viability was evaluated in HCT116 **A.** and SW480 **B.** CRC cells. Briefly, cells were grown, transfected with siRNA for *MEK1* and *MEK2* or *PIK3CA* and cellular viability determined by MTS assay 72h after transfection. Representative effects of MEK1/2 or PI3K p110α downregulation on HCT116 cells are shown (**C**, original magnification: x100). Representative Western blot **D, F.** and corresponding quantification analysis **E, G.** of MEK1/2, PI3K p110α (D, E) and PI3K p110β (F, G) upon silencing of *MEK1* and *MEK2* or *PIK3CA* in HCT116 CRC cells. Briefly, cells were grown, transfected with siRNA for *MEK1* and *MEK2* or *PIK3CA* and protein extracted 72h after transfection. Protein levels were assessed by Western blot analysis and subsequent quantification was performed. Controls included cells transfected with transfection reagent (mock ctrl) and Non-targeting siRNA. Data represent means ± SEM of at least triplicate experiments normalized to controls. All conditions were compared with Non-targeting siRNA. Ctrl, control. **, p< 0.01;****, p< 0.0001.

In this study, we have specifically tested the inhibition of *PIK3CA* that codes for PI3K p110α, the catalytic subunit of PI3K that is frequently mutated in CRC patients. Small interfering RNA technology was used as a first approach to assess how down-regulation of PI3K p110α could modulate cellular responses in CRC cells, ultimately impairing aberrant cell signaling. For this purpose, cellular viability was first investigated in HCT116 and SW480 CRC cells. Moreover, and in addition to PI3K p110α, inhibition of MEK1/2, a protein kinase downstream of KRAS and BRAF in the MAPK pathway, was also evaluated for comparison. Interestingly, and as demonstrated in Figure 1, downregulation of PI3K p110α significantly decreased cellular viability in HCT116 (Figure 1A) and SW480 (Figure 1B) cancer cell lines, regardless of the genetic alterations in *KRAS* and/or *PIK3CA*. In particular, the inhibition of PI3K p110α in HCT116 cells induced an almost 50% decrease in cellular viability (Figure 1A, p< 0.0001). Notably, this inhibitory effect in HCT116 cells was readily detectable under the microscope as shown in Figure 1C. In SW480 cells, the decline in cell viability was also remarkable as demonstrated in Figure 1B (p< 0.01). These results suggest that PI3K p110α plays a major role in mediating the survival of these CRC cell lines. In contrast, cell viability was not significantly reduced upon MEK1/2 inhibition in either HCT116 (Figure 1A) and SW480 (Figure 1B) cells (p> 0.05). Validation of successful inhibition of PI3K p110α and MEK1/2, upon targeted depletion of *PIK3CA* and *MEK1/2* by siRNA, was evaluated by Western blot. As shown in Figure 1D-1G, the siRNA knockdown approach for *PIK3CA* and *MEK1/2* was effective and selective. Indeed, HCT116 cells transfected with siRNA for *PIK3CA* and *MEK1/2* effectively inhibited PI3K p110α and MEK1/2 protein expression levels, respectively (Figure 1D–1E, p< 0.0001). Similar results were observed for SW480 (Supplementary Figure S1A-S1B, p< 0.01). Moreover, the use of *PIK3CA* siRNA did not affect the protein expression levels of PI3K p110β, a PI3K family member ubiquitously expressed, thus confirming selective down-regulation of PI3K p110α in these cell lines (Figure 1F–1G and Supplementary Figure S1C-S1D). Taken together, these results strongly support that PI3K p110α is essential to maintain viability of HCT116 and SW480 CRC cells.

### Specific inhibition of PI3K p110α induces apoptosis in *KRAS/PIK3CA* mutant cells and cell cycle arrest in *KRAS* mutant cells

**Figure 2:**
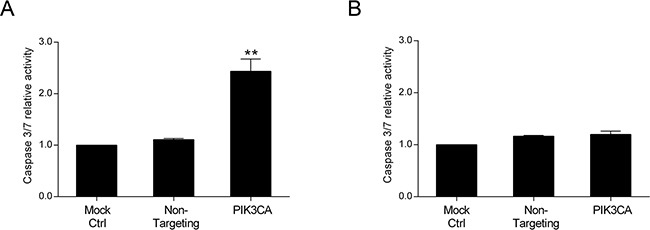
Inhibition of PI3K p110α induces apoptosis in HCT116 CRC cells The effect of siRNA-mediated targeted depletion of *PIK3CA* on apoptosis was evaluated by measuring the activity of caspases 3 and 7 in HCT116 **A.** and SW480 **B.** CRC cells. Briefly, cells were grown, transfected with *PIK3CA* siRNA and apoptosis determined by Caspase-Glo 3/7 assay (Promega) 72h after transfection. Controls included cells transfected with transfection reagent (mock ctrl) and Non-targeting siRNA. Data represent means ± SEM of at least triplicate experiments normalized to controls. *PIK3CA* siRNA was compared with Non-targeting siRNA. Ctrl, control. **, p< 0.01.

Our results indicate that inhibition of PI3K p110α highly impacts on cell viability in CRC cell lines with mutations in *KRAS* and/or *PIK3CA*. Thus, to define the molecular mechanisms underlying the effects of PI3K p110α inhibition, CRC cells were examined for both the levels of apoptosis and cell cycle stage. For this purpose, a caspase 3/7 activity assay which directly correlates with apoptosis, and propidium iodide staining for subsequent cell cycle analysis through flow cytometry were used. As shown in Figure 2, the results demonstrate that targeted depletion of *PIK3CA* resulted in a significant induction of caspase 3/7 activity and therefore an induction of apoptosis in *KRAS/PIK3CA* mutant HCT116 cells (Figure 2A, p< 0.01). However, no significant effect in apoptosis was observed in the *KRAS* mutant SW480 cells upon PI3K p110α inhibition (Figure 2B, p> 0.05).

**Figure 3:**
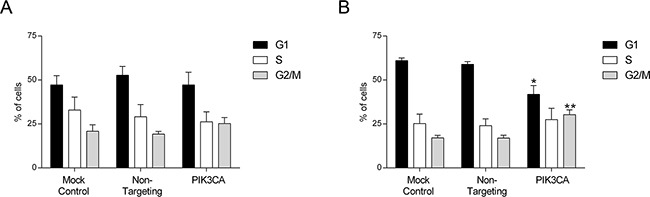
Inhibition of PI3K p110α induces cell cycle arrest in SW480 CRC cells The effect of siRNA-mediated targeted depletion of *PIK3CA* on cell cycle was evaluated by propidium iodide (PI) in HCT116 **A.** and SW480 **B.** CRC cells. Briefly, cells were grown, transfected with *PIK3CA* siRNA and PI staining performed 72h after transfection. Subsequent analysis was achieved by flow cytometry. Controls included cells transfected with transfection reagent (mock ctrl) and Non-targeting siRNA. Data represent means ± SEM of at least triplicate experiments. *PIK3CA* siRNA was compared with Non-targeting siRNA. Ctrl, control. *, p< 0.05; **, p< 0.01.

Conversely, as demonstrated in Figure 3, a shift in the cell cycle was observed upon PI3K p110α inhibition in *KRAS* mutant SW480 cells (Figure 3B) but not in *KRAS/PIK3CA* mutant HCT116 cells (Figure 3A). More specifically, inhibition of PI3K p110α in SW480 cells induced an arrest at the G2/M phase of the cell cycle reflected by an increase in the number of cells at G2/M from approximately 17% in the Non-targeting siRNA control to 30% in the *PIK3CA* siRNA (Figure 3B, p< 0.01). Concomitantly, a reduction in the number of cells at G1 phase from approximately 59% in the Non-targeting siRNA control to 42% in the *PIK3CA* siRNA was observed (Figure 3B, p< 0.05). In contrast, the proportion of cells in the S phase was not altered in these CRC cells (Figure 3B, p> 0.05). Overall, our data indicates that PI3K p110α inhibition induces apoptosis in *KRAS/PIK3CA* mutant HCT116 cells and a cell cycle arrest in *KRAS* mutant SW480 cells, suggesting that different mechanisms may be involved in mediating the inhibitory effects of PI3K p110α in CRC cell lines with distinct mutations, namely those in *KRAS* and *PIK3CA*.

### Apoptotic- and cell cycle-related proteins are altered upon PI3K p110α inhibition

**Figure 4:**
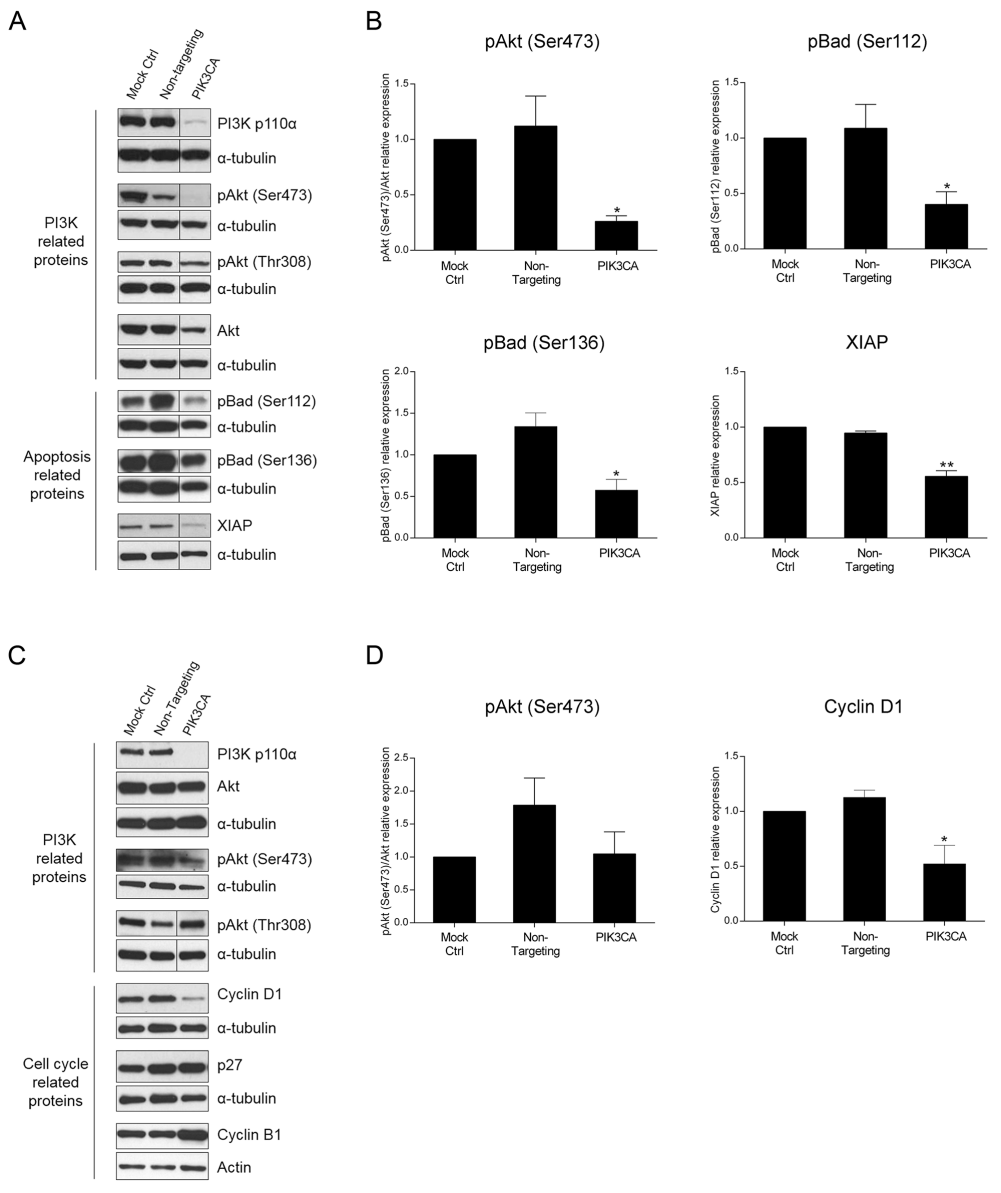
Inhibition of PI3K p110α induces downregulation of apoptotic- and cell cycle-related proteins in HCT116 and SW480 CRC cells, respectively Representative Western blot **A, C.** and corresponding quantification analysis **B, D.** of PI3K-, apoptosis- and cell cycle-related proteins upon silencing of *PIK3CA* in HCT116 (A, B) and SW480 (C, D) CRC cells. Briefly, cells were grown, transfected with *PIK3CA* siRNA and protein extracted 72h after transfection. Controls included cells transfected with transfection reagent (mock ctrl) and Non-targeting siRNA. Protein levels were assessed by Western blot analysis and subsequent quantification was performed. To improve clarity, Western blots were cropped when appropriate as indicated by vertical lines. Data represent means ± SEM of at least triplicate experiments normalized to controls. *PIK3CA* siRNA was compared with Non-targeting siRNA. Ctrl, control.*, p<0.05; **, p< 0.01.

The PI3K signaling cascade signals through a vast array of molecules downstream of PI3K to induce a specific cellular response. To identify the molecular targets underlying the effects of PI3K inhibition, we investigated downstream targets of PI3K in HCT116 and SW480 cells. More specifically, we evaluated members of the classical PI3K/Akt/mTOR pathway as well as PI3K targets involved in cell proliferation and survival, apoptosis and cell cycle. By Western blot analysis, we examined Akt and its phosphorylated (activated) forms pAkt (Ser473) and pAkt (Thr308). As demonstrated in Figure 4, knockdown of PI3K p110α significantly lowered the levels of pAkt (Ser473) in HCT116 cells (Figure 4A–4B, p< 0.05). Additionally, apoptotic-associated targets such as XIAP, pBad (Ser112) and pBad (Ser136) as well as cell cycle-associated targets including p27, cyclin B1 and cyclin D1 were also analyzed. Consistently with the results obtained for the caspase 3/7 activity, a significant alteration in the phosphorylated levels of the pro-apoptotic protein Bad was observed for the *KRAS/PIK3CA* mutant HCT116 cells. More specifically, a significant reduction of pBad (Ser112) and pBad (Ser136) protein expression was induced (Figure 4A–4B, p< 0.05) which is associated with Bad activation and promotion of apoptosis. Furthermore, and also in support of apoptosis induction, the levels of the anti-apoptotic protein XIAP significantly declined following PI3K p110α inhibition (Figure 4A–4B, p< 0.01). In contrast, pAkt (Thr308) (Figure 4A, p> 0.05) and cell cycle-related proteins (Supplementary Figure S2A, p> 0.05) did not differ dramatically upon PI3K p110α knockdown.

Next, we aimed to determine if PI3K- and cell cycle-related proteins were altered in SW480 cells. Indeed, although no significant decrease was observed on pAkt (Ser473) protein levels upon PI3K p110α inhibition, the results confirmed that the inhibition of PI3K p110α in SW480 cells induced a significant decline in cyclin D1, corroborating the results previously obtained (Figure 4C–4D, p< 0.05). The protein levels of cyclin B1 and p27 (Figure 4C, p> 0.05) as well as of apoptotic-related proteins (Supplementary Figure S2B, p> 0.05) were also evaluated, though no significant differences were observed. These results corroborate the data previously obtained and further suggest that different mechanisms are involved in mediating the PI3K p110α inhibitory effects in CRC cells with distinct mutations in *KRAS* and *PIK3CA*.

### The PI3K p110α inhibitor BYL719 mimics the effects of PIK3CA siRNA in *KRAS* and *KRAS/PIK3CA* mutant CRC cells

**Figure 5:**
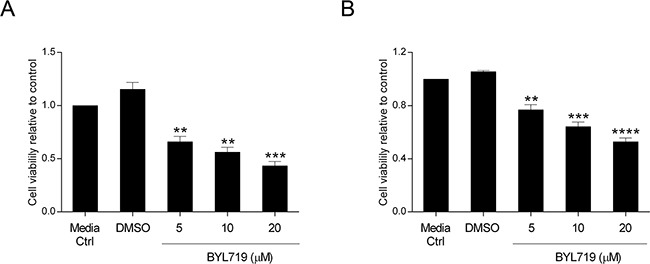
The selective PI3K p110α inhibitor BYL719 decreases viability in HCT116 and SW480 CRC cells The effect of BYL719 on cellular viability was evaluated in HCT116 **A.** and SW480 **B.** CRC cells. Briefly, cells were grown, treated with increasing concentrations of BYL719 (5, 10 and 20 μM) and cellular viability determined by MTS assay 72h after treatments. Controls included cells that remained untreated (media ctrl) and vehicle-treated controls (DMSO). Data represent means ± SEM of at least triplicate experiments normalized to controls. All conditions were compared with DMSO. Ctrl, control; DMSO, dimethyl sulfoxide. **, p< 0.01; ***, p< 0.001; ****, p< 0.0001.

**Figure 6:**
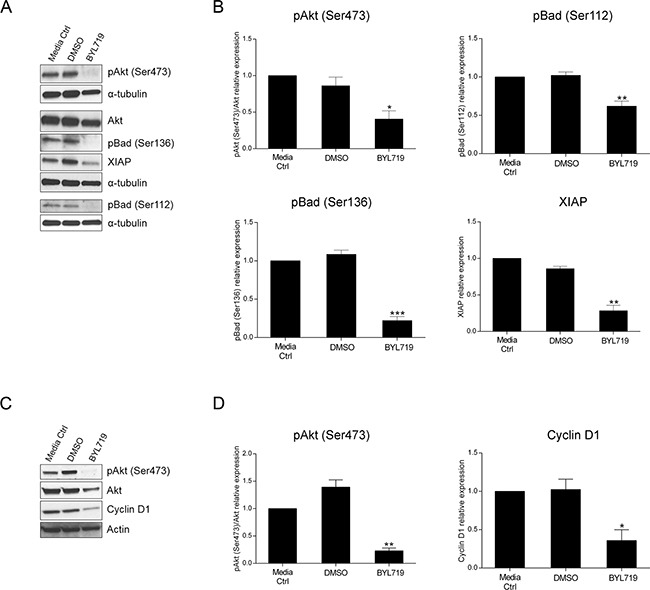
The selective PI3K p110α inhibitor BYL719 induces downregulation of apoptotic- and cell cycle-related proteins in HCT116 and SW480 CRC cells, respectively Representative Western blot **A, C.** and corresponding quantification analysis **B, D.** of PI3K-, apoptosis- and cell cycle-related proteins upon BYL719 treatment in HCT116 (A, B) and SW480 (C, D) CRC cells. Briefly, cells were grown, treated with 20 μM BYL719 and protein extracted 72h after treatments. Controls included cells that remained untreated (media ctrl) and vehicle-treated controls (DMSO). Protein levels were assessed by Western blot analysis and subsequent quantification was performed. Data represent means ± SEM of at least triplicate experiments normalized to controls. BYL719 treatment was compared with DMSO. Ctrl, control; DMSO, dimethyl sulfoxide. *, p< 0.05; **, p< 0.01; ***, p< 0.001.

Over the last few years, a multitude of drugs have been developed that target signaling components downstream of EGFR which are regarded as good candidates for therapeutic intervention. BYL719 is a selective PI3K p110α inhibitor already in clinical trials for many malignancies. Having validated the impact of PI3K p110α inhibition on CRC cell survival by siRNA, we sought to verify whether the same could be observed using a specific drug. Herein, we evaluated cellular viability after treatment with BYL719 (Selleck) in both HCT116 and SW480 CRC cell lines. Indeed, BYL719 induced a significant decline in cellular viability in HCT116 and SW480 cells in a dose dependent manner (Figure 5A–5B). More specifically, BYL719, was able to cause a decrease of more than 60% in the cellular viability of HCT116 cells (Figure 5A, p< 0.001) and a reduction of 50% in SW480 cells (Figure 5B, p< 0.0001) at the highest concentration used. Moreover, analysis of PI3K downstream targets revealed that inhibition of PI3K p110α leads to inhibition of downstream Akt signaling, as reflected by the significantly lower levels of pAkt (Ser473) in HCT116 (Figure 6A–6B, p< 0.05) and SW480 cells (Figure 6C–6D, p< 0.01). Furthermore, and mimicking the data previously obtained, we confirmed that BYL719 induced a significant decline in the levels of apoptotic-related proteins (Figure 6A–6B) including pBad (Ser112) (p< 0.01), pBad (Ser136) (p< 0.001) and XIAP (p< 0.01) in HCT116 cells and a decline in cyclin D1 protein levels (Figure 6C-6D, p< 0.05) in SW480 cells. Apoptosis- and cell cycle-related proteins were also evaluated in SW480 and HCT116 cells, respectively but no significant alterations were observed upon PI3K p110α inhibition (Supplementary Figure S3).

These results support the data obtained when using the *PIK3CA* siRNA approach and, indeed, both strategies confirm that PI3K p110α plays a major role in mediating the survival of CRC cell lines with *KRAS* and *PIK3CA* mutations, though by distinct mechanisms. Our data suggests that additional studies should be performed for the use of specific PI3K p110α inhibitors in CRC patients, particularly those with *KRAS* mutations who are currently excluded from the biological EGFR-targeted therapies.

## DISCUSSION

Colorectal cancer patients, particularly those with *KRAS* mutations, are still awaiting successful alternative targeted therapies to be implemented in clinical practice. Indeed, while CRC patients with wild type *KRAS* are eligible for targeted therapies with the anti-EGFR antibodies cetuximab or panitumumab, patients harboring *KRAS* mutations are excluded as mutations in *KRAS* are considered a predictive marker of resistance [14, 15]. In the last few years, a multitude of inhibitors that target a vast array of signaling molecules have emerged [11, 24, 27]. Among those inhibitors are drugs that target components of the PI3K signaling pathway, namely those inhibiting the various PI3K isoforms but regrettably these were shown to have limited clinical success [24, 28]. Therefore, our motivation was to overcome this constraint and test the inhibition of PI3K p110α as a therapeutic approach since the activation of this specific isoform has been implicated in CRC progression and *KRAS* mutants are able to signal through this pathway [4, 29].

Notably, studies evaluating PI3K signaling pathway inhibitors have also explored whether mutations, namely in *KRAS,* could be used as predictive biomarkers. However, and regrettably, *KRAS* mutations have been associated with resistance to targeted therapies involving PI3K/Akt/mTOR inhibitors, both in preclinical and clinical settings [24, 30–33]. In contrast, although preclinical data have suggested PI3K pathway alterations to be predictive of anti-tumor activity in response to PI3K/Akt/mTOR inhibitors, no consensus was yet attained in early clinical trials [30, 32–35]. In particular, Ihle *et al* reported that *RAS* mutations were considered predictors of anti-tumor activity resistance in human tumor xenografts treated with the PI3K inhibitor PX-866 [35]. Moreover, Di Nicolantonio *et al* demonstrated that cells with *PIK3CA* mutations had a distinct response to the mTOR inhibitor everolimus depending on the *KRAS* status. Indeed, cells with *PIK3CA* mutations but wild-type for *KRAS* were sensitized to this drug as opposed to mutant *KRAS* cells [30]. Other preclinical studies also indicated that coexistent mutations of *KRAS* and *PIK3CA* in CRC cells conferred resistance to the dual PI3K/mTOR inhibitor BEZ235 [31]. Importantly, in a cohort of metastatic cancer patients, *KRAS* mutations were associated with lack of benefit upon everolimus therapy [30]. Furthermore, in the particular case of CRC, patients with *PIK3CA* and *KRAS* mutations did not respond to the PI3K/Akt/mTOR axis therapy regimens [32, 33].

Thus, accumulating evidence has shown that CRC patients harboring *KRAS* mutations are still lacking successful treatments and await novel therapeutic strategies to be unraveled. Due to this disappointing scenario, we have investigated the impact of PI3K p110α specific inhibition on CRC cells with *KRAS* mutations. Our initial strategy involved the use of siRNA in an attempt to circumvent the “off-target” effects possibly associated with small molecule inhibitors. In this context, our results have shown that inhibition of *PIK3CA* by siRNA in CRC cell lines has a major impact in cellular viability, not only in cells with mutations in *KRAS/PIK3CA* but also in those with *KRAS* mutations i.e. our data indicates that CRC cells with *KRAS* mutations are sensitive to PI3K p110α inhibition, regardless of *PIK3CA* concomitant mutations. These results could indicate that the constitutive activation of KRAS in CRC cells with *KRAS* mutations could signal, at least partially, through PI3K. Indeed, binding of RAS to p110α has been shown to be required for tumor induced angiogenesis and RAS-driven tumorigenesis in mice [36, 37].

Interestingly, we observed that the functional responses to the specific PI3K p110α inhibition were distinct depending on the genetic background for *KRAS* and *PIK3CA*. In particular, *PIK3CA* silencing in CRC cells harboring *KRAS/PIK3CA* mutations induced apoptosis, as demonstrated by the increased levels of caspase 3/7 activity. In contrast, a shift in the cell cycle phase was observed in CRC cells harboring *KRAS* mutations, more specifically, the inhibition of PI3K p110α induced an arrest at G2/M and a concomitant reduction in the number of cells at G1 phase.

As above mentioned, it is well established that the PI3K signaling pathway is involved in regulating a vast array of proteins, namely proliferation, survival and apoptosis proteins, ultimately modulating key cellular processes [20, 21]. Thus, to further explore the mechanisms involved in mediating the PI3K p110α inhibitory effects, a number of downstream targets were investigated in this study. We showed that Akt signaling, a classical PI3K downstream signaling, was altered upon PI3K p110α inhibition by siRNA as demonstrated by the decrease in the levels of pAkt (Ser473) in *KRAS/PIK3CA* CRC mutant cells. Furthermore, we analyzed proteins as pBad and XIAP which are considered apoptotic-related proteins as well as cell cycle regulation proteins as p27, cyclin B1 and cyclin D1. Notably, and supporting the increase in apoptosis levels previously observed in CRC cells with *KRAS/PIK3CA* mutations, PI3K p110α inhibition by siRNA induced alterations in two apoptosis-related proteins, pBad and XIAP [38, 39]. In particular, a significant reduction of both pBad (Ser112) and pBad (Ser136) was observed in HCT116 cells leading to Bad activation and subsequently to apoptosis. The anti-apoptotic protein XIAP is a known inhibitor of caspases, namely of caspases 3 and 7 [39]. Remarkably, XIAP protein levels also decreased upon PI3K p110α inhibition, once again indicative of apoptosis induction, further supporting our data on caspase 3/7 activity.

Interestingly, in *KRAS* mutant CRC cells our data demonstrates that cyclin D1 protein levels were significantly downregulated upon PI3K p110α inhibition by siRNA. Notably, cyclin D1 is an established regulator of cell cycle progression well known to be involved in the G1/S phase transition [40]. However, recent reports have shown that cyclin D1 is also implicated in G2 induction, namely in the context of RAS-dependent mitosis progression [41, 42] and downregulation of cyclin D1 has been associated with cell cycle arrest in G2 upon oxidative stress [43]. Indeed, our data on cyclin D1 further supports our results and shows a reduction in the number of cells at G1 and an arrest at G2/M.

More recently, selective inhibitors of PI3K isoforms have emerged [24]. Nevertheless, the mechanisms underlying the inhibitory responses are still not well understood, namely in the context of CRC with mutations in *KRAS*. Therefore, and taking into consideration the above mentioned results, we tested the recently developed PI3K p110α inhibitor, BYL719. BYL719, a 2-aminothiazole derivative, was developed on the basis of a binding model to 2-aminothiazole compounds and although the exact mechanism of action is yet to be understood, BYL719 is currently under clinical evaluation for many cancer types including CRC (NCT01719380) [24, 25, 44]. In preclinical studies, BYL719 was shown to have anti-tumor activity in many cancer cell types namely nasopharyngeal, head and neck and osteosarcoma cells and recent evidence suggested *PIK3CA* mutant cells to be more sensitive to BYL719 [26, 45–47]. Our results showed that BYL719 was able to induce a significant decline in the viability of CRC cells with *KRAS* and *KRAS/PIK3CA* mutations. Moreover, and mimicking the siRNA data, BYL719 was able to modulate cell cycle- and apoptosis-related proteins. Specifically, BYL719 downregulated cyclin D1 protein levels in CRC cells with *KRAS* mutations as well as pBad (Ser112), pBad (Ser136) and XIAP in *KRAS/PIK3CA* mutant CRC cells, further validating the siRNA data.

Altogether, our data demonstrates that inhibition of PI3K p110α has an impact in SW480 and HCT116 CRC cells that harbor mutations in *KRAS* and *KRAS/PIK3CA*, respectively. Indeed, not only cells with *PIK3CA* mutations were sensitive to PI3K p110α inhibition but also cells with *KRAS* mutations, emphasizing the complexity and interconnection of the signaling pathways. Notably, distinct mechanisms were shown to be involved in mediating the effects of PI3K p110α inhibition in the *KRAS* and *KRAS/PIK3CA* mutant CRC cells which could be explained, at least in part, by the coexistence of *KRAS* and *PIK3CA* mutations but also by other features including the type of *KRAS* mutation (HCT116, *KRAS*^G13D^; SW480, *KRAS*^G12V^). Indeed, coexistence of *KRAS* and *PIK3CA* mutations as well as the type of *KRAS* mutations in CRC cells have been associated with different outcomes upon drug treatment [30, 31, 48]. Moreover, HCT116 and SW480 cells, which are routinely used in preclinical studies, have distinct alterations namely in terms of microsatellite instability and gene mutations as in *p53*, a well-known regulator of apoptosis and cell cycle arrest [49–51]. Notably, in contrast to p53 mutant SW480 cells, in p53 wild type HCT116 cells apoptosis was induced upon PIK3 p110α inhibition suggesting that an intact p53 could be required for the apoptotic pathway. Overall, these alterations could also contribute to the distinct cellular responses. Nonetheless and most remarkably, *KRAS* mutant CRC cells, which correspond to the subset of patients who are currently excluded from EGFR targeted therapies, were sensitive to PI3K p110α inhibition regardless of the additional alterations.

In the future, it will be important to evaluate PI3K p110α inhibition in CRC cells with alternative mutational patterns including: distinct hotspot mutations in *KRAS* and *PIK3CA*, *NRAS* mutations, *BRAF* mutations as well as wild type *KRAS*. In addition, combination of PI3K p110α inhibition with other targeted therapies, namely anti-EGFR antibodies, could provide alternative and improved therapeutic strategies that would be particularly interesting in the context of drug resistance. Indeed, acquired resistance is still a major problem and remains a challenge in cancer therapy development. Recent data in breast cancer cells have shown that BYL719 resistance was conferred by IGF1R/p110β/Akt/mTOR activation [52]. Notably, deregulation of IGF2 signaling leading to activation of IGF1R was reported in CRC which could also be relevant in the context of PI3K p110α inhibition and combined therapeutic interventions [53].

In conclusion, our data has awarded PI3K p110α a key role in CRC cells, namely those with *KRAS* mutations. This is of particular relevance, as patients harboring *KRAS* mutations are currently excluded from EGFR-targeted therapies. We provided compelling evidence supporting PI3K p110α as a good target for therapeutic intervention particularly in CRC with *KRAS* mutations. Further insights into the available PI3K p110α drugs and a better understanding of the underlying mechanisms will help to improve this therapeutic strategy which will ultimately have an impact in the clinical management, and eventually, on the overall survival rate of CRC patients.

## MATERIALS AND METHODS

### Cell culture and chemicals

Human CRC cell lines harboring hotspot mutations in KRAS and/or PIK3CA were used in this study, more specifically HCT116 (KRAS^G13D^, PIK3CA^H1047R^) and SW480 (KRAS^G12V^). Briefly, cells were grown in RPMI-1640 with L-glutamine and HEPES (Gibco, Invitrogen) supplemented with 10% (v/v) fetal bovine serum (FBS, Hyclone) and 1% (v/v) penicillin/streptomycin (Gibco, Invitrogen). All cells were grown in a humidified incubator at 37°C, 5% CO_2_. Microscope images were acquired using an inverted Nikon ECLIPSE TS100 microscope with a 10x/0,25 objective (Nikon) equipped with a DS camera control unit DS-L2 (Nikon). The PI3K p110α inhibitor BYL719 was purchased from Selleck Chemicals LLC (Houston, USA) and a stock solution of 10mM in dimethyl sulfoxide (DMSO) was used. For BYL719 experiments, cells were seeded, allowed to attach overnight and treated with 5, 10 or 20 μM BYL719 for 72h. Controls included cells that remained untreated (media control) and vehicle-treated controls (DMSO).

### Gene silencing by siRNA

Small interfering RNA (siRNA) for *PIK3CA, MEK1* and *MEK2* were purchased from Dharmacon. Specifically, ON-TARGETplus SMARTpool for *PIK3CA* (L-003018-00), *MEK1* (L-003571-00) and *MEK2* (L-003573-00) were used. ON-TARGETplus Non-targeting pool (Dharmacon, D-001810-10) was also included as a control. Briefly, cells were seeded and allowed to attach overnight. Transfections were then performed with *MEK1, MEK2* and *PIK3CA* siRNA (50 or 100 nM) or Non-targeting siRNA with DharmaFECT 2 Transfection reagent (Dharmacon) according to the manufacturer's recommendations. All subsequent assays were performed 72h following transfections. Controls included cells transfected with transfection reagent (mock control) and Non-targeting siRNA.

### Protein extraction and western blot analysis

Whole cell protein extracts were prepared in radioimmunoprecipitation assay (RIPA) lysis buffer (1% NP-40 in 150 mM NaCl, 50 mM Tris (pH 7.5), 2mM EDTA) supplemented with protease (Roche) and phosphatase inhibitors (Sigma). Protein concentration was determined using a Bradford assay kit (Bio-Rad). For Western blot analysis, protein samples (15-25 μg) were resolved on 10% sodium dodecyl sulphate (SDS)-polyacrylamide gel electrophoresis (PAGE) or on 4-20% Mini protean TGX gradient gels (Bio-Rad) under denaturing conditions and transferred to Hybond ECL membranes (Amersham Biosciences). Primary antibodies for PI3K p110α (#4249), pAkt (Ser473) (#4060), pAkt (Thr308) (#2965), Akt (#9272), MEK1/2 (#4694), cyclin D1 (#2926), cyclin B1 (#12231), p27 (#3686), pBad (Ser112) (#5284) and pBad (Ser136) (#4366) were all purchased from Cell Signalling whereas antibodies for XIAP (#ab113089), α-tubulin (#T5168) and actin (#Sc-1616) were from Abcam, Sigma and Santa Cruz Biotechnology, respectively. Secondary antibodies, goat anti-rabbit and goat anti-mouse, were conjugated with horseradish peroxidase (Santa Cruz Biotechnology) and detected with ECL reagent (Amersham). Actin or α-tubulin were used as loading controls. Representative Western blots are shown and in some cases, to improve clarity, Western blots were cropped as depicted by a vertical line and indicated in the figure legend. Quantification of the specific signals was performed using Quantity One software (Bio-Rad). All proteins were normalized to the corresponding loading control. In addition, pAkt (Ser473) and pAkt (Thr308) were normalized to total Akt. Data represent means ± SEM of at least triplicate experiments normalized to controls.

### Cell viability assay

The CellTiter 96 AQueous One Solution Cell Proliferation Assay (MTS, Promega) was used as an indicator of cell viability. Briefly, cells were seeded in 96-well plates and transfected with siRNA or treated with BYL719, as previously specified. For the MTS assay, cells were then incubated with the MTS reagent as specified by the manufacturer's instructions and absorbance read at 490 nm in a plate-reader. Data represent means ± SEM of at least triplicate experiments normalized to controls.

### Caspase 3/7 activity

The Caspase-Glo 3/7 Luminescent assay (Promega), that measures caspase 3 and 7 activities, was used as an indicator of apoptosis. Briefly, cells were seeded in white walled multi 96-well plates and transfected with siRNA as previously specified. For the caspase 3/7 assay, cells were incubated with Caspase-Glo 3/7 reagent as specified by the manufacturer's instructions, and luminescence read in a plate-reading luminometer. Data represent means ± SEM of at least triplicate experiments normalized to controls.

### Flow cytometry

Cell cycle analysis was performed using propidium iodide (PI) staining followed by flow cytometry. Briefly, cells were platted in 6-well plates and transfected with siRNA as above mentioned. For the PI assay, cells were trypsinized, washed in phosphate-buffered saline (PBS) solution and fixed in 70% ethanol. Cells were then washed twice in PBS and stained for 1 h with 50 μg/mL PI (Invitrogen) containing 100 μg/ml DNase-free RNase A (Qiagen). For cell cycle evaluation, DNA content was assessed by flow cytometry using a FACS Calibur (BD Biosciences) and data analysed using the FlowJo 7.6.5 software. Data represent means ± SEM of at least triplicate experiments.

### Statistical analysis

Data are presented as mean values ± SEM of at least triplicate experiments. Statistical analysis was performed using GraphPad Prism (version 6.05) and the unpaired two-tailed t-test was used for comparison of parameters between two groups. A p value < 0.05 was considered statistically significant. Values statistically significant were depicted as following: *, p< 0.05; **, p< 0.01; ***, p< 0.001; ****, p< 0.0001.

## SUPPLEMENTARY MATERIALS FIGURES


