# Supplementary material for: Specific inhibition of p110α subunit of PI3K: putative therapeutic strategy for KRAS mutant colorectal cancers
Source: Oncotarget. 2016 Sep 2;7(42):68546–58. doi: 10.18632/oncotarget.11843 (PMC5356572; doi:10.18632/oncotarget.11843)
Supplement: Supplementary file 1 [file oncotarget-07-68546-s001.pdf]

Specific inhibition of p110α subunit of PI3K: putative therapeutic strategy for *KRAS* mutant colorectal cancers

SUPPLEMENTARY FIGURES

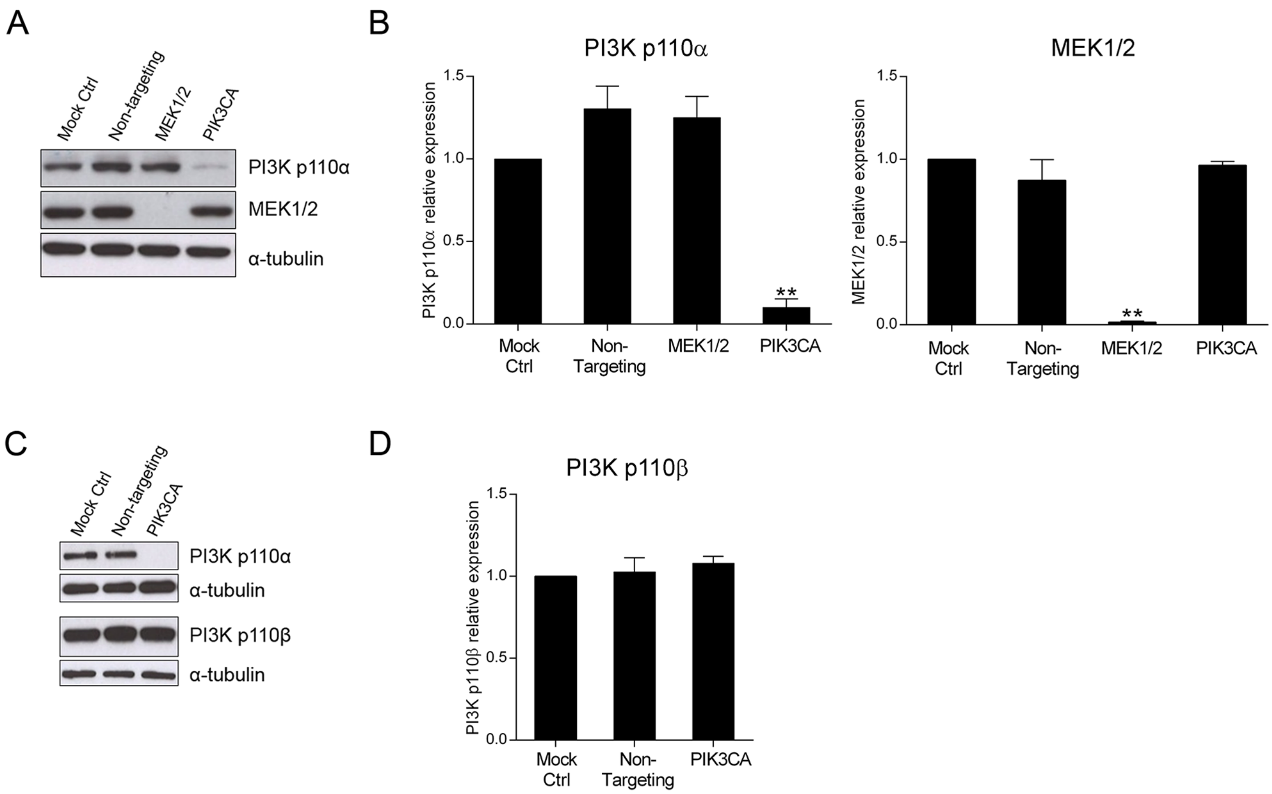

Supplementary Figure S1: Silencing of *PIK3CA* and *MEK1/2* in SW480 CRC cells downregulates PI3K p110α and MEK1/2 protein expression, respectively. Representative Western blot **A, C.** and corresponding quantification analysis **B, D.** of MEK1/2, PI3K p110α (A, B) and PI3K p110β (C, D) upon silencing of *MEK1* and *MEK2* or *PIK3CA* in SW480 CRC cells. Briefly, cells were grown, transfected with siRNA for *MEK1* and *MEK2* or *PIK3CA* and protein extracted 72h after transfection. Protein levels were assessed by Western blot analysis and subsequent quantification was performed. Controls included cells transfected with transfection reagent (mock ctrl) and Non-targeting siRNA. Data represent means ± SEM of at least triplicate experiments normalized to controls. All conditions were compared with Non-targeting siRNA. Ctrl, control. **, $p< 0.01$.

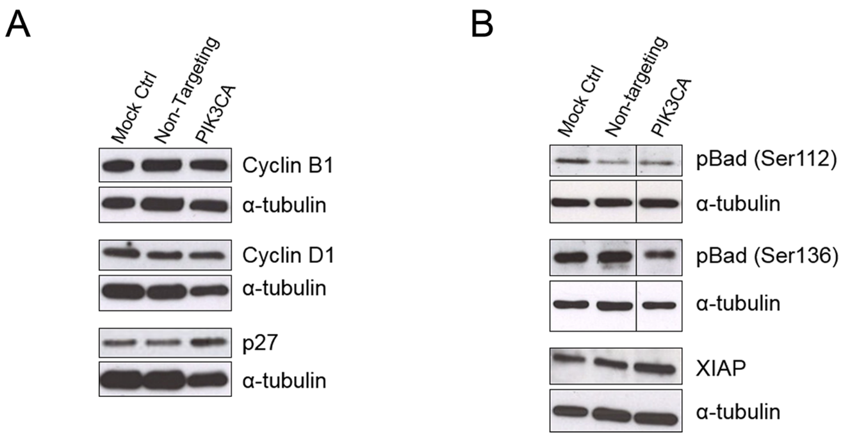

Supplementary Figure S2: Effect of the inhibition of PI3K p110α by siRNA in cell cycle- and apoptotic-related proteins in HCT116 and SW480 CRC cells, respectively. Representative Western blot of cell cycle-related proteins in HCT116 cells **A.** and apoptosis-related proteins in SW480 cells **B.** upon silencing of *PIK3CA*. Briefly, cells were grown, transfected with *PIK3CA* siRNA and protein extracted 72h after transfection. Controls included cells transfected with transfection reagent (mock ctrl) and Non-targeting siRNA. To improve clarity, Western blots were cropped when appropriate as indicated by vertical lines. Ctrl, control.

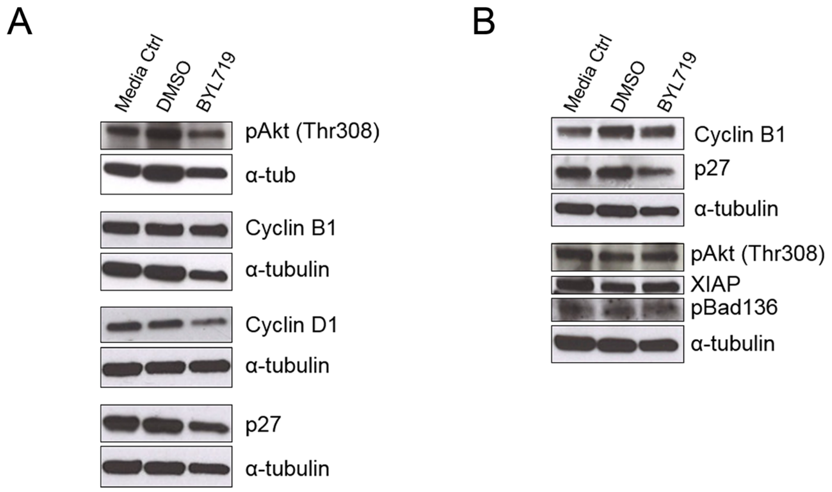

Supplementary Figure S3: Effect of the selective PI3K p110α inhibitor BYL719 in PI3K-, cell cycle- and apoptotic-related proteins in HCT116 and SW480 CRC cells. Representative Western blot of PI3K-, cell cycle- and apoptosis-related proteins in HCT116 **A.** and SW480 **B.** CRC cells upon BYL719 treatment. Briefly, cells were grown, treated with 20 µM BYL719 and protein extracted 72h after treatments. Controls included cells that remained untreated (media ctrl) and vehicle-treated controls (DMSO). Ctrl, control; DMSO, dimethyl sulfoxide.
